# Healthcare access barriers for Hispanic pediatric nephrology patients: a KICK study

**DOI:** 10.1007/s00467-025-06881-4

**Published:** 2025-07-12

**Authors:** Debora Matossian, Carlos C. Becerril Romero, Priya S. Verghese

**Affiliations:** Ann and Robert H. Lurie Children’s Hospital, 225 E. Chicago Ave Box 37, Chicago, IL USA

**Keywords:** Pediatrics, Hispanic, Barriers, Chronic, Kidney, Spanish

## Abstract

**Background:**

Hispanics, the fastest growing minority in the USA, suffer from high chronic kidney disease (CKD) burden. Barriers to accessing medical care increase disparities and quality of care for Hispanic children with CKD.

**Methods:**

As a part of the grant-funded “Kidney Initiative in Community Kids” (KICK), the voluntary “Barriers to Care Questionnaire” was distributed to self-identified Hispanic young adult patients (> 18 years of age) or parents (if patient < 18 years of age), between January 2021 to June 2021, in a single urban pediatric nephrology ambulatory clinic site. Survey was available in English and Spanish. Sociodemographic and health data was obtained from the medical record and Area Deprivation Index (ADI) was collected as a surrogate marker for socioeconomic disadvantage.

**Results:**

Of the 179 completed surveys (49% Spanish, 51% English), skills (strategies necessary to navigate the healthcare system) and pragmatic (logistical issues that might delay/prevent utilization) barriers were identified with mean (± standard deviation) scores of 74 (± 26) and 75 (± 21), respectively (100 = no barriers). Spanish-speaking preference families had higher skills barriers (*p* < 0.001). The mean ADI was 55 ± 20: higher ADI score (more disadvantaged population) correlated with higher total barriers (*p* = 0.04) and was exacerbated by preferred language (*p* = 0.003).

**Conclusions:**

Hispanic pediatric patients with kidney disease have significant barriers which may impact their interaction with healthcare. Spanish-speaking families are particularly vulnerable. Prospective studies are necessary to advocate for program and policy changes to reduce racial and ethnic disparities in access to pediatric nephrology care.

**Graphical abstract:**

**Figure:**
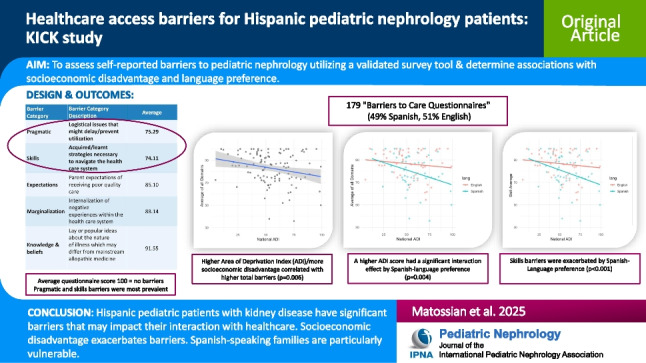
A higher resolution version of the Graphical abstract is available as Supplementary information

**Supplementary Information:**

The online version contains supplementary material available at 10.1007/s00467-025-06881-4.

## Introduction

Hispanics are the fastest-growing minority in the USA [1], with the largest population of youth 0–19 years of age. Hispanics are at increased risk for chronic kidney disease (CKD) and CKD progression due to genetic non-modifiable risk factors and modifiable risk factors related to social determinants of health, such as poverty, food insecurity, and obesity [2]. For example, adult data from the Hispanic chronic renal insufficiency study shows that 63% of Hispanics with CKD have incomes less than $20,000 compared to 16% of non-Hispanic Whites, and 23% of Hispanics with CKD have no insurance compared to 6% of non-Hispanic Whites [3]. A large percentage of the pediatric Hispanic population similarly resides in very low-opportunity neighborhoods where access to healthy, affordable food is limited or non-existent. There is a high rate of food insecurity at 33% in Hispanic children [2] and food insecurity is associated with obesity [4]. Hispanic children have the highest rates of obesity in all age groups regardless of gender [4, 5], with rates as high as 50% in some under-resourced neighborhoods. Obesity is closely linked with and can lead to the progression of CKD through mechanisms associated with glucose intolerance, dyslipidemia, hypertension, and atherosclerosis [6]. The COVID-19 pandemic has intensified existing health disparities associated with food insecurity. The increased rate and risk of progression of CKD in Hispanic patients is compounded by a low kidney transplant rate [7]. Pediatric Hispanic patients are 42% less likely than non-Hispanic Whites to receive a living donor transplant [8]. The existence of health disparities in access, quality, and outcomes in the Hispanic population is not new, and is well documented [9]. However, specifics on self-identified mechanisms of barriers to healthcare in the Hispanic pediatric population are poorly defined. Barriers to care, or socio-behavioral processes that hinder a family’s ability to engage positively with the healthcare system, can decrease the chances of timely access, productive interactions, and favorable outcomes [10]. Barriers to care may be an important target for programs; barriers need to be defined and quantified. In this study, as part of our Kidney Initiative in Community Kids (KICK), we assess self-reported barriers to pediatric subspecialty care utilizing a validated survey tool called Barriers to Care Questionnaire and determined associations with socioeconomic disadvantage and language in an urban pediatric nephrology center.

## Methods

### Barriers assessment

Between January through June 2021, all patients at Lurie Children’s Hospital of Chicago who self-identified as Hispanic in the electronic medical record (EMR) were provided with a validated survey tool, “Barriers to Care Questionnaire (BCQ)” [10]. The survey was given to the patient or caregiver at check-in for their routine pediatric nephrology appointments by the front desk staff. Participants were requested to complete it on paper while waiting to be roomed. The survey was filled out by parents/legal guardians unless the patients were older than 18 years, in which case they would fill it out themselves. Completion of the survey was voluntary. Forms were offered in Spanish or English. The survey had 40 questions divided into five categories:

1. Pragmatic: [9 questions] these identified logistical issues that might delay/prevent utilization of healthcare.
2. Skills: [8 questions] questions attempted to capture information on acquired/learnt strategies that would be necessary to navigate the healthcare system.
3. Marginalization: [11 questions] Questions in this category were designed to understand the internalization of negative experiences within the healthcare system.
4. Expectations: [7 questions] this section asked questions on parent expectations of receiving poor quality care.
5. Knowledge and Belief: [4 questions] patients were asked questions to better understand their ideology (lay or popular ideas) about the nature of their child’s illness, and whether it differed from mainstream allopathic medicine.

### Health and sociodemographic data collection

We collected sociodemographic and health data from the electronic medical record. Sociodemographic data included age, gender, race, primary and secondary diagnosis, number of medications taken, distance to travel from home to hospital in miles, insurance, zip code, and adherence to stipulated clinic follow-up. The Area Deprivation Index (ADI), a measure of socioeconomic disadvantage, which incorporates 17 socioeconomic indicators was collected. The national ADI ranking ranges from 1 (most advantaged) to 100 (most disadvantaged). The ADI is based on a measure developed by the Health Resources and Services Administration (HRSA) which has been refined, adapted, and validated at the Census block group neighborhood level [11]. Health data included blood pressure by the updated 2017 American Academy of Pediatrics guidelines’ classification [12], body mass index (BMI) categorization (underweight, healthy, overweight, obese), height (cm) and weight (kg) with their corresponding percentiles, total cholesterol (mg/dl), serum glucose (mg/dl), hemoglobin A1C %, serum creatinine (mg/dl), serum cystatin C (mg/L), estimated glomerular filtration rate (eGFR) by Schwartz, pediatric CKID calculator, or CKD-EPI adult calculator if older than 18 years old, urine protein to creatinine ratio, and 25 hydroxy vitamin D level (ng/ml).

### Statistical plan

We used descriptive analysis to characterize sociodemographic, health, and survey responses. Barriers to care were summarized based on point scale. Each item of the survey was transformed to a 100-point scale (100, 75, 50, 25, 0) with 100 equating to no barriers and any score below 75 equating a “fail,” indicating unacceptably high barriers. Mean scores for all categories were separated by preferred language (language chosen to complete survey) and two sample t-test was run to determine if the difference was significant. The survey results were not normally distributed; therefore, non-parametric tests were used. Spearman’s correlation was utilized to assess relationships between numeric variables. Kruskal and Mann–Whitney were used to test differences in test scores and other numeric variables across groups. We created linear regression models to describe the association between ADI and survey scores and tested for interactions based on language preference.

## Results

### Survey administration

Patients who self-identified as Hispanic in the EMR were approached with survey (*N* = 615). Of the 615 patients screened during the study period, 179 patients completed the survey (29% completion rate). Fifty-one percent preferred to answer the survey in English and 49% answered the survey in Spanish. One hundred nine patients provided identifying information (date of birth/name/medical record number). There was a significant association (*p* = 0.003) between the language the survey was taken in and identifying information collected. Half of the Spanish surveys did not include identifying info (50.7%), compared to just under 30% for English (29.3%). Survey respondents were most often parents/guardians (88%) and in some cases, the patient themselves were > 18 years of age and completed the survey independently (12%).

### Study population, sociodemographic and health parameters

Health and demographic information was collected on the 109 patients who provided identifying information on surveys. The average patient age of the cohort was 12 years old (range 1–21 years) with a slight male predominance (54%). Most patients had public insurance (76%), 43% travelled > 20 miles with 2 patients (2%) living more than 100 miles from the clinic (Table 1). The primary etiology that prompted their nephrology visit varied: CKD in 36%, hypertension in 26%, and kidney transplant in 14%. The stage of CKD varied: 57% had stage 1, 29% stage 2, 9% stage 3, 4% stage 4, 1% stage 5 (Table 2). The mean eGFR was normal or near normal depending on the equation used (Schwartz 102 ml/min/1.73 m^2^, CKiD with Cystatin C 82 ml/min/1.73 m^2^). Patients were taking an average of 4 different types of medications. Metabolic syndrome parameters including average lipid and glucose levels were normal but the average hemoglobin A1c (5.7%) was in the pre-diabetes range (5.7–6.4%). Most of the cohort was overweight (63%), and only one patient was underweight.

**Table 1:** Sociodemographic

Variable	Totals (*n* = 109)
Age (years)	12.03 (std 5.21)
Gender	Male (*n* = 59, 53.2%)
	Female (*n* = 50, 45.9%)
Race	White (*n* = 18, 16.7%)
	Black (*n* = 3, 2.8%)
	Other (*n* = 87, 80.6%)
Language of survey	English (*n* = 56, 51.4)
	Spanish (*n* = 53, 48.6%)
Primary diagnosis	CKD (*n* = 42, 38.5%)
	Hypertension (*n* = 27, 24.8%)
	Kidney Transplant (*n* = 15, 13.8%)
	Other (*n* = 25, 22.9%)
Number of medications	4.26 (std 4.72)
No show at follow-up	17 (16.2%)
Insurance	Public (*n* = 83, 76.9%)
	Other (*n* = 25, 23.1%)
Travel distance to clinic	0–10 miles (*n* = 31, 28.4%)
	10–20 (*n* = 32, 29.4%)
	20–100 (*n* = 44, 40.4%)
	Over 100 (*n* = 2, 1.8%)

**Table 2:** Clinical and laboratory parameters

Variable, units	Means, SD
Total cholesterol, mg/dl	150.70 (39.33)
High density lipoprotein, mg/dl	48.20 (16.44)
Low density lipoprotein, mg/dl	76.00 (30.25)
Glucose, mg/dl	95.26 (21.63)
25 OH vitamin D, ng/ml	27.59 (11.36)
Hemoglobin A1C, %	5.74 (1.46)
BMI, percentile	77.66 (28.03)
BMI kg/m^2^	24.87 (7.29)
Systolic blood pressure, mmHg	113.16 (13.93)
Diastolic blood pressure, mmHg	72.04 (10.27)
eGFR Schwartz, ml/min/1.73 m^2^	102.00 (33.42)
eGFR CKiD, ml/min/1.73 m^2^	82.40 (19.57)
eGFR adult with creatinine, ml/min/1.73 m^2^	96.47 (45.02)
eGFR adult with creatinine and cystatin C, ml/min/1.73 m^2^	105.70 (26.63)
Serum creatinine, mg/dl	0.80 (0.82)
Serum cystatin, mg/l	1.07 (0.36)
Urine protein to creatinine ratio	0.35 (0.56)
CKD severity	1 – 54 (57.45%)
	2 – 27 (28.72%)
	3 – 8 (8.51%)
	4 – 4 (4.26%)
	5 – 1 (1.06%)

*BMI* body mass index, *eGFR* estimated glomerular filtration rate, *CKD* chronic kidney disease

### Identified barriers in study population

**Fig. 1:**
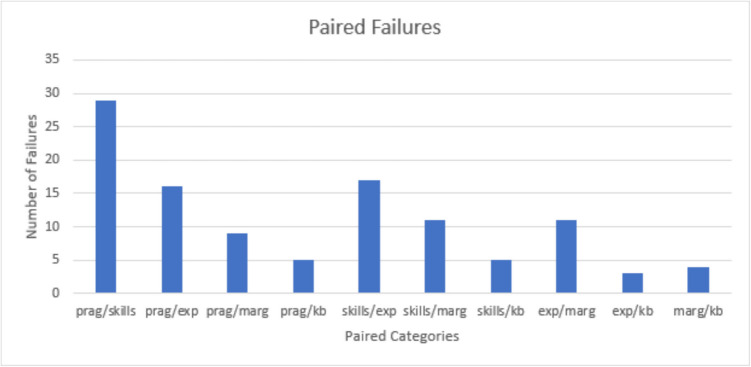
Abbreviations: Pragmatic (prag); Expectations (exp); Knowledge and Beliefs (kb); Marginalization (marg)

Barriers to care were highly prevalent in the categories of Skills and Pragmatic: mean (± standard deviation) scores of 74 (± 26) and 75 (± 21) respectively (Table 3). Sixty-one percent of participants failed at least 1 category, 40% failed 2 categories, 21% failed 3 categories, and 10% failed 4 categories. Participants who failed Pragmatic often failed Skills as well. Barriers in Knowledge and beliefs were rare with a mean score of 92 (±), and when present, this barrier did not occur in isolation (Fig. 1).

**Table 3:** Barriers to Care Questionnaire results by category. [Scores range 0–100 with 100 indicating no barriers in that category, and a score below 75 equating a “fail”

Category	Category Interpretation	Average (± SD)	Total failures/denominator (%)
Pragmatic	Logistical issues that might delay/prevent utilization	75.29 (20.65)	45/109 (41)
Skills	Strategies necessary to navigate the healthcare system	74.11 (26.14)	42/108 (38)
Expectations	Parent expectations of receiving poor quality care	85.10 (19.12)	23/108 (21)
Marginalization	Internalization of negative experiences within the healthcare system	88.14 (16.78)	16/104 (15)
Knowledge/Beliefs	Lay or popular ideas about the nature of illness which may differ from mainstream allopathic medicine	91.55 (15.15)	6/103 (5.8)

### Impact of language and area deprivation index

**Fig. 2:**
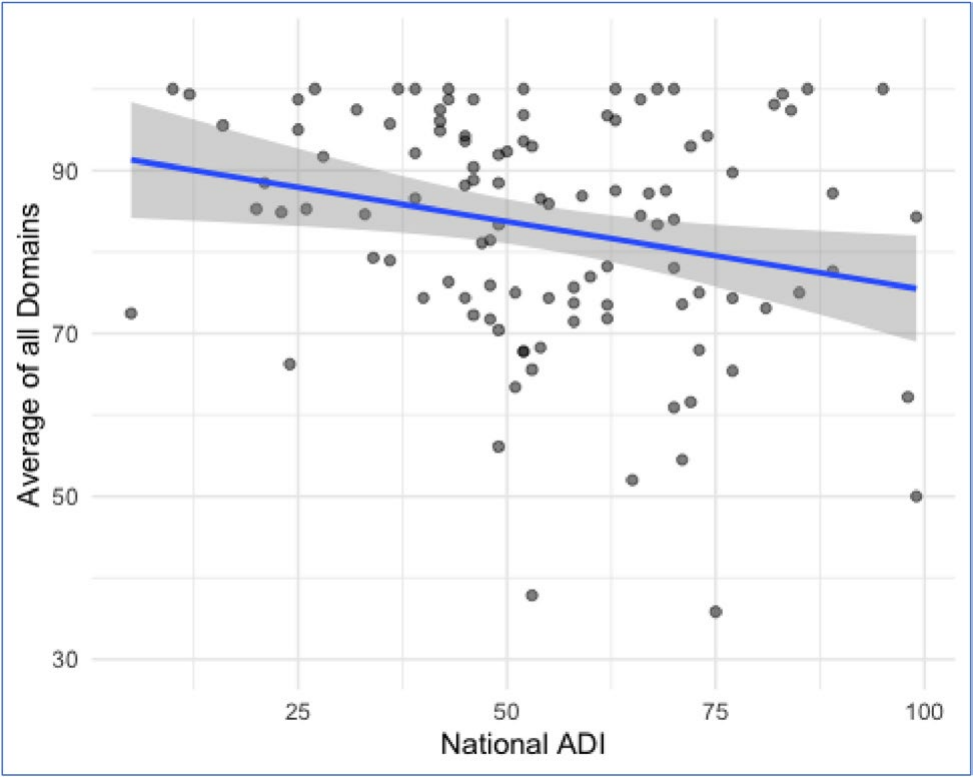
Average total score compared to National Area Deprivation Index (ADI), all participants

**Fig. 3:**
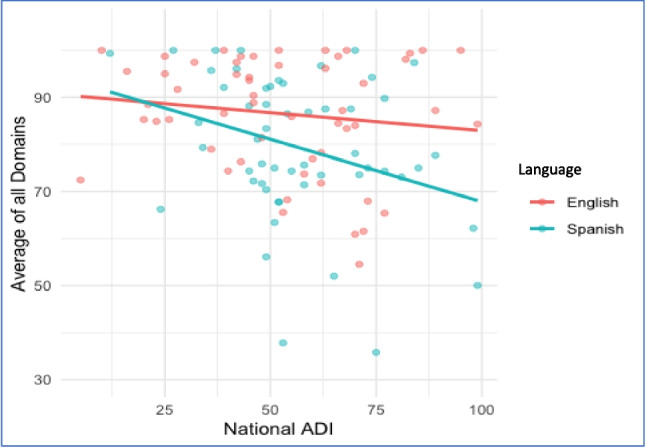
Interaction between language and Area Deprivation Index (ADI)

**Fig. 4:**
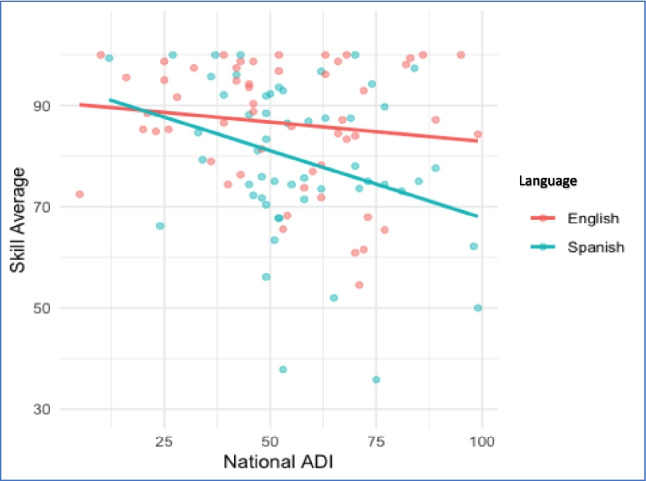
Average Skill score by National ADI, by primary language

The mean national ADI score was 55 (± 20). A higher ADI score correlated with higher total barriers (*p* = 0.006) (Fig. 2). A higher ADI score had a significant interaction effect by Spanish-language preference (*p* = 0.04) (Fig. 3). There was no difference in Pragmatic barriers based on language preference (*p* = 0.4). Pragmatic barriers remained high among English 77 (± 21) and Spanish speakers 74 (± 21). Conversely, Skills barriers were exacerbated by Spanish-language preference (*p* < 0.001) (Fig. 4). Spanish speakers had more skills barriers 64 (± 28) compared to English speakers 84 (± 120).

## Discussion

Our study demonstrates significant barriers to care in Hispanic children with pediatric nephrology subspecialty needs in an outpatient, urban setting. To our knowledge, this has not been done before. Barriers were frequently identified in the categories of Skills and Pragmatism. Pragmatic barriers could include challenges like travelling to the doctor’s office, reaching the office by phone, waiting too long for an appointment, accessing care after hours or on the weekends, managing household responsibilities, taking time off work, enduring long waits in the waiting room, meeting the needs of other family members, and dealing with the cost of healthcare. Barriers related to Skills might include knowing how to navigate the healthcare system, dealing with doctors or nurses who are not fluent in your language or use overly technical medical language, getting referrals to specialists, understanding doctor’s instructions, having insufficient information about how the healthcare system operates, needing to be more knowledgeable to access healthcare, or struggling to get help with paperwork [10].

Spanish-language preference was associated with barriers related to information learnt to navigate the medical system (i.e., Skills barriers). This is intuitive since healthcare familiarity and skill acquirement are achieved through direct and indirect exposure, and the primary language at healthcare institutions is English. Raising awareness of the communication barriers, enhancing communication skills with the help of translators, and spending more time with patients may improve communication and trust. Incentive pay for fluency, a distinctive skill that benefits the patient, could also deserve further consideration [13]. Spanish-language families struggle to communicate effectively with care providers and therefore likely fail to advocate for their children’s health. Others have demonstrated that children whose parents’ primary language is not English are more likely to be underdiagnosed [14] and have more problems accessing or using the medical system [15]. Our findings give us a direct actionable goal towards building a Spanish-speaking workforce which could help improve communication and reduce skill barriers for vulnerable non-English preference patients and families. This is particularly relevant since 19.5% of the USA today are primarily Spanish-speaking, but only 5% of Pediatric Nephrologists in the USA identified as Hispanic [16]. Enhancing workforce representation could help empower families to navigate the medical system with fewer barriers.

While we did not collect data on parental education, immigration status, or income information for fear of the patient perception of stigmatization, instead, we used the Area of Deprivation Index (ADI) as a surrogate for socioeconomic disadvantage. This tool incorporates factors related to the theoretical domains of income, education, employment, and housing quality. When factoring ADI, we found that Spanish-language preference was associated with a higher socioeconomic disadvantage, and greater socioeconomic deprivation exacerbates barriers to care. We suspect Spanish-language preference likely correlates with more recent immigration, less education, or salaried jobs, and difficulties in performing the necessary tasks recommended by medical professionals and thus decreased health outcomes.

In our study, barriers in the Knowledge and Beliefs category were the least commonly identified. Hispanics share a strong heritage that includes family and religion as the leading players in decision-making, concluding this finding as surprising. Furthermore, a study in adults to provide insights into how Hispanic communities perceive living kidney donation found the opposite, that lack of knowledge and misconceptions were the most significant barriers for Hispanics regarding living donor kidney transplantation [17]. Perhaps, our findings represent that adults, though influenced by non-mainstream medicine for themselves, trust and abide by the physician’s recommendations for their children. Whether this is due to intrinsic or extrinsic factors, not limited to the role that child protective services play in the USA, cannot be determined in this study. Despite our finding, we emphasize the importance of a diverse workforce, multicultural care provider teams, and cultural congruency to support Spanish-language families to navigate the medical system [18].

Our clinical-anthropometric data is consistent with that previously reported in the adult Hispanic population, with 63% of patients being overweight or higher, and the average hemoglobin A1C in the pre-diabetes range. It is a reminder for ongoing attention to modifiable risk factors like obesity and diabetes in the Hispanic pediatric population to reduce the risk of CKD development and progression. A contemporaneous assessment of our kidney transplant population yielded that 19.5% of the sample were identified as food insecure, and our Hispanic patients had higher food insecurity compared to non-Hispanics (56% vs. 30%, *p* = 0.015) [manuscript under review]. The association between food insecurity and higher BMI in Hispanic youth has been reported [9], and obesity is closely linked with and can lead to CKD progression [6, 19]. We speculate that Hispanic immigrant families have lower access to government-based supplemental nutrition programs, likely secondary to legal status, fear of deportation, poverty, or language barriers. Lack of access to healthy, fresh food is likely exacerbating a modifiable risk factor for suffering obesity, CKD, and a higher rate of disease progression.

Study limitations include selection bias, as the individuals who completed the survey may not represent the broader population of Hispanic patients receiving pediatric nephrology care. Furthermore, the study relies on self-reported data, which can introduce biases such as recall bias or social desirability bias. Although the survey was available in both English and Spanish, there may still be language comprehension issues, especially among those who are not fully fluent in either language. The study might not account for variations in literacy levels or nuances in language understanding, which could influence how participants interpreted and answered the survey questions. The study was conducted at a single urban center in Chicago. This limits the external validity, as the results may not be applicable to Hispanic populations in other geographic regions, especially rural areas or locations with different socioeconomic conditions, healthcare access, or cultural contexts. Regarding use of the ADI, while it is a relevant measure, it oversimplifies the complex, multi-dimensional factors that contribute to healthcare barriers. Finally, the study only provides a snapshot of the barriers to care at a single point in time. This limits the ability to infer causal relationships or observe changes over time. Longitudinal data would be necessary to assess how barriers evolve and how they might influence health outcomes in the long term.

Despite our study’s limitations, it is still impactful as the survey completion rate was satisfactory with balanced representation of Spanish-speaking and English-speaking Hispanic patients. Approximately 42% of our center’s patient population is Hispanic; therefore, the study population is representative of the city-wide ethnic and language prevalence (Data from 7/2018 through 6/2022. Data from all Emergency Department, observation, and inpatient admissions to Ann and Robert H Lurie Children’s Hospital). Census data from 2021 states that 20.4% of the population in Chicago is foreign-born, and of these, the majority (51.8%) came from Spanish-speaking countries [20]. As of 2023, the USA is the second largest Spanish-speaking country in the world, with 57 million speakers, including both native speakers and those who speak Spanish as a second language [21].

The study’s use of voluntary participation and provision of surveys in both English and Spanish increases representation and demonstrates an ethical commitment to inclusivity. It addresses a significant and timely issue, highlighting the intersection of genetics, socioeconomic factors, and healthcare access, making it a valuable contribution to understanding and addressing inequities in healthcare outcomes for the Hispanic population. The statistical rigor and use of a validated survey tool ensures a robust and accurate measurement of the socio-behavioral factors affecting healthcare engagement and a holistic understanding of barriers. The study integrates a comprehensive set of socioeconomic data, including the ADI, therefore enabling targeted interventions with special focus on the significant role that language can play in accessing and navigating healthcare services. These strengths collectively enhance the study’s significance, offering valuable insights into healthcare disparities faced by Hispanic children with CKD, and laying the groundwork for future research and interventions to improve healthcare access and outcomes for this population.

Larger prospective studies are needed to comprehensively assess the barriers to subspecialty care and the impact of language preference faced by this growing ethnic group. We would also suggest additional studies to assess whether our findings can be extrapolated to other non-English-speaking patient populations.

## Conclusion

Our study demonstrates multiple barriers for urban Hispanic youth in accessing pediatric nephrology healthcare, including but not limited to socioeconomic status and language. Spanish-language preference represents a particularly vulnerable subgroup of Hispanics who face greater challenges in accessing quality care. Reducing health inequities requires a deep understanding for the growing diversity within the US pediatric population, as well as research into and recognition of the negative impact of various social determinants of child health. This is a call to the pediatric nephrology community for targeted interventions to increase equitable high-quality pediatric nephrology healthcare access for Hispanic patients.

## Supplementary information

Below is the link to the electronic supplementary material.

Graphical abstract (PPTX 1.38 MB)

Supplementary Material 1 (PDF 99.4 KB)

Supplementary Material 2 (PDF 167 KB)

## Data Availability

The data supporting the findings of this study are available upon request from the corresponding author, Debora Matossian, MD.
