# Supplementary material for: Healthcare access barriers for Hispanic pediatric nephrology patients: a KICK study
Source: Pediatr Nephrol. 2025 Jul 12;40(11):3477–83. doi: 10.1007/s00467-025-06881-4 (PMC12484322; doi:10.1007/s00467-025-06881-4)
Supplement: Supplementary file 1 — Graphical abstract (PPTX 1.38 MB) [file 467_2025_6881_MOESM1_ESM.pptx]

## Slide 1
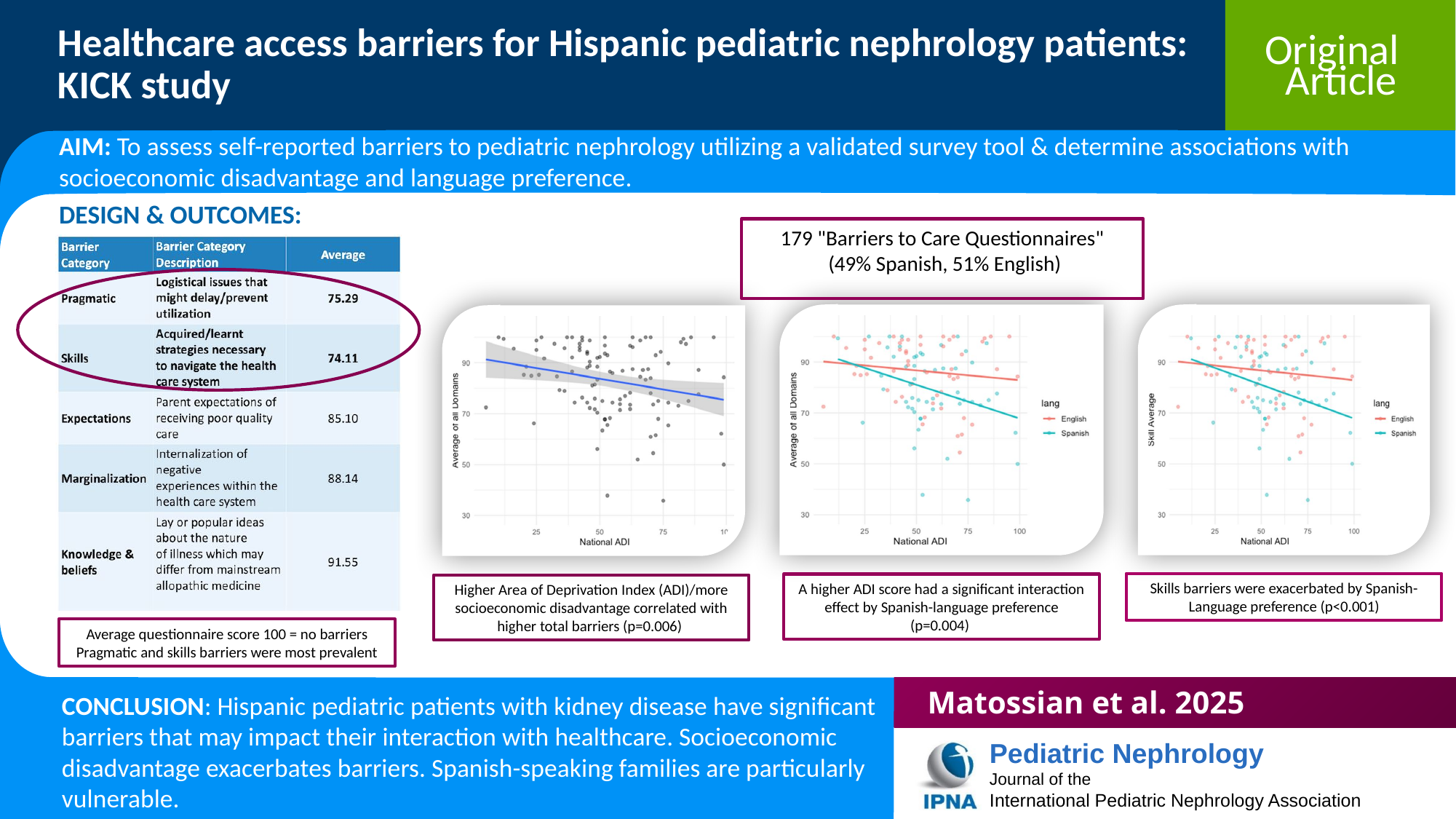

Healthcare access barriers for Hispanic pediatric nephrology patients: KICK study
AIM: To assess self-reported barriers to pediatric nephrology utilizing a validated survey tool & determine associations with socioeconomic disadvantage and language preference.
DESIGN & OUTCOMES:
179 "Barriers to Care Questionnaires"
 (49% Spanish, 51% English)
Skills barriers were exacerbated by Spanish-Language preference (p<0.001)
A higher ADI score had a significant interaction effect by Spanish-language preference (p=0.004)
Higher Area of Deprivation Index (ADI)/more socioeconomic disadvantage correlated with higher total barriers (p=0.006)
Average questionnaire score 100 = no barriers
Pragmatic and skills barriers were most prevalent
Matossian et al. 2025
CONCLUSION: Hispanic pediatric patients with kidney disease have significant barriers that may impact their interaction with healthcare. Socioeconomic disadvantage exacerbates barriers. Spanish-speaking families are particularly vulnerable.
