# Supplementary material for: Healthcare access barriers for Hispanic pediatric nephrology patients: a KICK study
Source: Pediatr Nephrol. 2025 Jul 12;40(11):3477–83. doi: 10.1007/s00467-025-06881-4 (PMC12484322; doi:10.1007/s00467-025-06881-4)
Supplement: Supplementary file 2 — Supplementary Material 1 (PDF 99.4 KB) [file 467_2025_6881_MOESM2_ESM.pdf]

Problems getting health care for my child

Parents often face barriers when trying to get health care for their children. We are interested in the kinds of things that interfere with getting health care for your child(ren). Please rate how much of a problem each of the following is for you.

Answer each question by completely shading the circle so that it looks like this: ●

		Never	Almost Never	Sometimes	Often	Almost Always
1.	In the last 3 months, how often did the health care system work well for your child?	(0)	(1)	(2)	(3)	(4)

How often were each of the following barriers a problem in the past 3 months when trying to get health care for your child:

**Problems with:**		Never	Almost Never	Sometimes	Often	Almost Always
2.	Getting to the doctor's office	(0)	(1)	(2)	(3)	(4)
3.	Getting hold of the doctor's office or clinic by phone	(0)	(1)	(2)	(3)	(4)
4.	Having to wait too many days for an appointment	(0)	(1)	(2)	(3)	(4)
5.	Getting care after hours or on the weekends	(0)	(1)	(2)	(3)	(4)
6.	Having to take care of household responsibilities	(0)	(1)	(2)	(3)	(4)
7.	Having to take time off work	(0)	(1)	(2)	(3)	(4)
8.	Having to wait too long in the waiting room	(0)	(1)	(2)	(3)	(4)
9.	Knowing how to make the health care system work for you	(0)	(1)	(2)	(3)	(4)
10.	Meeting the needs of other family members	(0)	(1)	(2)	(3)	(4)
11.	The cost of health care	(0)	(1)	(2)	(3)	(4)
12.	Doctors or nurses not fluent in your language	(0)	(1)	(2)	(3)	(4)
13.	Doctors or nurses who speak in a way that is too technical or medical	(0)	(1)	(2)	(3)	(4)
14.	Getting referrals to specialists	(0)	(1)	(2)	(3)	(4)
15.	Understanding doctor's orders	(0)	(1)	(2)	(3)	(4)
16.	Having enough information about how the health care system works	(0)	(1)	(2)	(3)	(4)
17.	Needing to be more 'savvy' or knowledgeable about getting health care	(0)	(1)	(2)	(3)	(4)
18.	Getting enough help with paperwork or forms	(0)	(1)	(2)	(3)	(4)
19.	Offices and staff that are not child-friendly	(0)	(1)	(2)	(3)	(4)
20.	Mistakes made by doctors or nurses	(0)	(1)	(2)	(3)	(4)
21.	Worrying that doctors and nurses will not do what is right for your child	(0)	(1)	(2)	(3)	(4)

Problems with:	Never	Almost Never	Sometimes	Often	Almost Always
22. Doctors treating the symptom without finding out the cause of the illness	(0)	(1)	(2)	(3)	(4)
23. Getting a thorough examination	(0)	(1)	(2)	(3)	(4)
24. Lack of communication between my child's doctor and others in the health care system	(0)	(1)	(2)	(3)	(4)
25. Lack of communication between different parts of the health care system	(0)	(1)	(2)	(3)	(4)
26. Feeling like doctors are trying to give as little service as possible	(0)	(1)	(2)	(3)	(4)
27. Feeling like the health care system is trying to give as little service as possible	(0)	(1)	(2)	(3)	(4)
28. Impatient doctors	(0)	(1)	(2)	(3)	(4)
29. Intimidating doctors	(0)	(1)	(2)	(3)	(4)
30. Rude office staff	(0)	(1)	(2)	(3)	(4)
31. Uncaring office staff	(0)	(1)	(2)	(3)	(4)
32. Getting the doctor to listen to you	(0)	(1)	(2)	(3)	(4)
33. Getting your questions answered	(0)	(1)	(2)	(3)	(4)
34. Not knowing what to expect from one visit to the next	(0)	(1)	(2)	(3)	(4)
35. Being judged on your appearance, your ancestry, or your accent	(0)	(1)	(2)	(3)	(4)
36. Doctors rushing you and your child through the visit	(0)	(1)	(2)	(3)	(4)
37. Disagreeing with the doctor's orders	(0)	(1)	(2)	(3)	(4)
38. Doctors not believing in home or traditional remedies	(0)	(1)	(2)	(3)	(4)
39. Doctors giving you instructions that seem wrong	(0)	(1)	(2)	(3)	(4)
40. Doctors or nurses that have different ideas about health than you do	(0)	(1)	(2)	(3)	(4)
