# Supplementary material for: Healthcare access barriers for Hispanic pediatric nephrology patients: a KICK study
Source: Pediatr Nephrol. 2025 Jul 12;40(11):3477–83. doi: 10.1007/s00467-025-06881-4 (PMC12484322; doi:10.1007/s00467-025-06881-4)
Supplement: Supplementary file 3 — Supplementary Material 2 (PDF 167 KB) [file 467_2025_6881_MOESM3_ESM.pdf]

Date: 11-18-2020

IRB #: IRB 2020-3640
Title: Community Health Needs Assessment for Chicago Youth, Adolescents and Families
Creation Date: 4-7-2020
End Date:
Status: Approved
Principal Investigator: Debora Matossian
Review Board: Panel #1
Sponsor:

Study History

Submission Type	Initial	Review Type	Limited	Decision	Exempt - Limited IRB
Submission Type	Modification	Review Type	Limited	Decision	Exempt - Limited IRB

Key Study Contacts

Member	Debora Matossian	Role	Principal Investigator	Contact dmatossian@luriechildrens.org
Member	Priya Verghese	Role	Co-Principal Investigator	Contact pverghese@luriechildrens.org
Member	Stephen Harris	Role	Primary Contact	Contact sharris@luriechildrens.org
Member	Sara Malik	Role	Investigator	Contact saramalik@luriechildrens.org

Initial Submission

1 General Information

Study Identification

Guidance for specific questions is included to the right of the question in the Helper Text (Question Mark Icon).

A

- Links to applicable sections of the IRB Policies & Procedures Manual are included throughout this application for reference.
- For more information about the IRB submission Process, IRB Tracking, and Cayuse IRB, please refer to the Cayuse IRB Submission Process guide.

What type of submission is this?

A.1

For details regarding the types of submission, please refer to IRB Policies & Procedures Manual Section 9.

Research Study Involving Human Subjects - **Expedited Review**
(Study involves procedures that are no more than minimal risk.)

Research Study Involving Human Subjects - **Full Board Review**
(Study involves procedure(s) greater than minimal risk or a minor increase over minimal risk; or the study includes an investigational device that is non-exempt and requires a Risk Determination.)

✓ Research Study Involving Human Subjects - **Exempt Determination Request**

Research Study Involving Human Subjects - **External IRB Review**
(i.e. request for Lurie Children's IRB to rely on an External IRB)

Treatment Use of Investigational Drug or Device

(i.e. Expanded Access, Humanitarian Use Device (HUD), Compassionate Use - Device)

Emergency Use of an Investigational Drug or Device

Use of Protected Health Information (PHI) Preparatory to Research

Case Report / Case Series

Quality Improvement / Quality Assurance Project

Use of Decedents' Protected Health Information (PHI)

Non-Human Subjects Research Determination

B Study Personnel

For guidance regarding PI Responsibilities and who to list in Study Personnel, refer to IRB Policies & Procedures Section 5.

B.1 Select the Principal Investigator (PI).

Any study conducted by a PI who is not a Lurie Children's employee must have at least one Lurie Children's faculty member within the division/department where the research will be conducted serve as a Sub-Investigator.

Name: Debora Matossian
Organization: Kidney Diseases
Address: , Chicago, IL 60611-2605
Phone: 312-227-6165
Email: dmatossian@luriechildrens.org

Select all Primary Study Contact(s).

B.2 Name: Stephen Harris
Organization: Kidney Diseases
Address: , Chicago, IL 60611-2605
Phone:
Email: sharris@luriechildrens.org

Select all Sub-Investigators.

B.3 Name: Priya Verghese
Organization: Users loaded with unmatched Organization affiliation.
Address: , Minneapolis, MN 55455-2070
Phone:
Email: pverghese@luriechildrens.org

Select all other study staff.

B.4

If you are working with REDCap at Northwestern, please list "REDCap User" in this role so that REDCap staff may access to approval status and letters.

B.5 Select non-Lurie Children's personnel engaged in the study conduct at Lurie Children's site(s).

B.6 To whom has the PI delegated responsibility to obtain informed consent?

- All Primary Study Contact(s) listed in B.2
- All Sub-Investigators listed in B.3
- All other study staff listed in B.4
- All non-Lurie Children's personnel listed in B.5
- Limited to the following personnel:
- ✓ Not applicable (waiver of informed consent being requested or informed consent not required)

Exempt Determination Request

J

For guidance regarding studies exempt from IRB review, refer to [IRB Policies & Procedures Manual Section 9.2.b].

J.1 Select all categories into which this study most closely fits:

Category 1:
This research will be conducted in an established or commonly accepted educational setting.

It will involve normal educational practices that are not likely to adversely impact students' opportunity to learn required educational content or the assessment of educators who provide instruction.

The study procedures include:

- research on regular and special education instructional strategies; **or**
- research on the effectiveness of the comparison among instructional techniques, curricula, or classroom management methods.

✓ **Category 2:**
This research only includes interactions involving one or more of the following:

- educational tests (cognitive, diagnostic, aptitude, achievement)
- survey procedures
- interview procedures
- observation of public behavior (including visual or auditory recording)

J.1a Please select the interaction(s) to be included in this study.

Educational tests (cognitive, diagnostic, aptitude, achievement, etc).

✓ Surveys and/or interviews.

J.1a-2 Confirm that children will not be included as subjects in this study.

✓ Yes

No

J.1a-3 Upload the surveys and/or interview scripts to be used for this study.

[BarriersSurveyEng.doc]

[BarriersSurveySpa.doc]

Observation of public behavior (including visual or auditory recording).

J.1b Studies may be exempt under this category if at least one of the following criteria is met. Select all that apply:

The information will be recorded so that the identity of the participant **cannot** readily be ascertained (i.e. the study data will not contain identifiers and will not be linked to identifiers via a code).

The disclosure of the participants' responses outside the research would not reasonably place the participants at risk of criminal or civil liability or be damaging to the participants' financial standing, employability, educational advancement, or reputation.

✓ The information obtained is recorded so that the identity of participant **can** readily be ascertained (i.e. the study data is recorded with identifiers or identifiers are linked via a code).

Note: Studies involving Northwestern University Feinberg School of Medicine students or Northwestern/McGaw residents or fellows as study participants are to be reviewed and approved by Diane Wayne, MD, Vice-Dean, Education, Northwestern University Feinberg School of Medicine (dwayne@northwestern.edu).

Category 3:

This research involves benign behavioral interventions.

- The information will be collected from an **adult** participant through verbal or written responses (including data entry) or audiovisual recording; and

- The subject will be asked to prospectively agree to the intervention and information collection (i.e. written or verbal consent will be sought).

Benign behavioral interventions are brief in duration, harmless, painless, not physically invasive, not likely to have a significant adverse lasting impact on the participants, and the investigator has no reason to think the participants will find the interventions offensive or embarrassing.

Category 4:

✓ The secondary research use of identifiable private information or identifiable biospecimens.

J.1e Studies may be exempt under this category if at least one of the following criteria is met. Select all that apply:

The identifiable private information or identifiable biospecimens are publicly available.

The information will be recorded so that the identity of the participant **cannot** readily be ascertained (i.e. the study data will not contain identifiers and will not be linked to identifiers via a code).

In addition, the investigator will not contact the participants and the investigator will not re-identify participants.

✓ The information obtained is recorded so that the identity of participant **can** readily be ascertained (i.e. the study data is recorded with identifiers or identifiers are linked via a code). For example, collecting identifiable or coded data from the medical record.

This research is being conducted by, or on behalf of, a Federal department or agency using government-generated or government-collected information obtained for nonresearch activities.

Notes: Studies involving Northwestern University Feinberg School of Medicine students or Northwestern/McGaw residents or fellows as study participants are to be reviewed and approved by Diane Wayne, MD, Vice-Dean, Education, Northwestern University Feinberg School of Medicine (dwayne@northwestern.edu).

Provide the source of the data and/or specimens:

J.1h
- For example: Lurie Children's medical records, leftover clinical biospecimens, a research repository or database, Centers for Medicare & Medicaid Services database, etc.).

Lurie Children's medical records

Category 5:
This research /demonstration project is being conducted or supported by a Federal department or agency, or it is subject to the approval of a specific Federal department or agency/bureau or other subordinate agencies that have been delegated authority to conduct this project.

This research is designed to study, evaluate, improve, or otherwise examine public benefit or service programs, including:

- procedures for obtaining benefits or services under those programs;
- possible changes in or alternatives to those programs or procedures; or
- possible changes in methods or levels of payment for benefits or services under those programs.

(Projects applicable for this category include, but are not limited to, internal studies by Federal employees, and studies under contracts or consulting arrangements, cooperative agreements, or grants.)

Category 6:
Taste and food quality evaluation and consumer acceptance studies:

- if wholesome foods without additives are consumed; **or**
- if a food is consumed that contains a food ingredient at or below the level and of a use found to be safe, or agricultural chemical or environmental contaminant at or below the level found to be safe, by the Food and Drug Administration (FDA) or approved by the Environmental Protection Agency (EPA) or the food Safety and Inspection Services of the U.S. Department of Agriculture (USDA).

Briefly describe the study being proposed and provide the background and justification of the study including appropriate references if not included in a separate document/protocol in Section 9.

The intervention proposed is to evaluate the pediatric Hispanic population we serve in the Division of Kidney Diseases at Lurie Children’s Hospital.

The purpose of our study is to:

a)

Do a cross-sectional analysis of the pediatric Hispanic patient population of the Division of Nephrology at Ann & Robert H. Lurie Children’s Hospital. By means of a retrospective chart review of our pediatric Hispanic patients, we will analyze demographics (including zip code analysis and surrogates for socio-economic status); obesity/overweight rates, rates of adherence/follow up, presence of hypertension, distribution of kidney conditions they suffer from, glomerular filtration rate, proteinuria (the term Hispanic refers to all persons of Latin American origin living in the United States, unless indicated otherwise)

b)

Administer a simple questionnaire for patient families to evaluate the status of access to care for Hispanics; identification of unique issues of this population that precludes optimal follow up and care.

The primary study outcomes will be:

(1) To identify risk factors (disease specific and demographic) for progression of CKD in the Hispanic population served in the Kidney Diseases Division at LCH

(2) To perform a survey analysis of reported difficulties and limitations towards adequate access to care

Data to be collected:

• Zip Code
• Preferred language
• Age
• Gender
• Race
J.2 • Ethnicity
• Food insecurity
• Transportation needs
• Social documentation
• Financial resource strain
• Insurance
• Number of clinic visits within the study period
• Number of telemed / telephone / in person visits Following implementation of the telemed program at Lurie’s
• Number of hospitalizations within the study period
• No show rate
• Body mass index percentile (BMI = ht. in m2/wt. in kg.) for age and gender;
• Height and weight measurements
• Height and weight percentiles
• Most recent outpatient clinic BPs
• Clinical diagnosis to require clinical follow up at the Division of Kidney Diseases (primary + 4 additional from problem list)
• Renal replacement (Hemodialysis, Peritoneal dialysis, Transplantation, kidney transplant waitlist)
• Concurrent serum cystatin C levels;

• Vitamin D levels;
• Serum glucose
• Total cholesterol, Triglycerides, HDL, LDL
• Hemoglobin A1C (if available)
• Urine protein/creatinine to calculate a ratio
• Serum creatinine
• Concurrent calculated eGFR

Survey:
-We will utilize a validated survey tool "Barriers to Care Questionnaire (BCQ)" in Spanish and English)\ to assess limitations to access to care.
-Survey will be completed by parents or patients older than 18 years old if they come by themselves.
-Paper survey tool will be handed to families at check-in.
- Survey will be voluntary.
-An information sheet will be attached to the survey.
- Survey will be placed in an envelope and will be collected by the research coordinator periodically.

List the inclusion and exclusion criteria for this study.

J.3

Inclusion criteria:
•Hispanic pediatric patients followed clinically in the division of kidney diseases at LCH
•Aged newborn to 21 years old

Exclusion criteria:
•Non-Hispanic patients

J.4

Provide the following:

- A plan to minimize any potential risks to participants,
- The process by which the privacy of participants and the confidentiality of the research data will be maintained; and
- A plan for monitoring the safety and welfare of enrolled participants.

All data will be stored in a secure password-protected database. All completed surveys will be stored in locked cabinets in locked offices.

Risk to subjects whose data will be collected is minimal. The primary risk is loss of confidentiality. As a retrospective chart review study, there is no plan for monitoring the safety and welfare of enrolled participants.

K Data Use/Disclosure

K.1 Will medical records or other sources of identifiable health information be accessed as a part of this proposal?

✓ Yes

K.1a Will the patient or patient's Legally Authorized Representative (LAR) be asked to sign a HIPAA Authorization Form for this proposed use of PHI?

Yes

✓ No - full waiver of HIPAA Authorization requested

K.1a-1 Confirm that the use or disclosure of PHI identifiers involves no more than minimal risk to the privacy of the participants.

✓ Yes

K.1a-2 Explain why this proposal cannot practicably be conducted without the Waiver of HIPAA Authorization to access and use the identifiers.

This is a retrospective review covering patients treated at Lurie Children's Hospital over a 3 year period.. If HIPAA authorization is required, it is likely that many patients would not be able to be contacted which would significantly negatively impact the quality of the data.

K.1a-3 Confirm that the PHI will be destroyed at the earliest opportunity.

✓ Yes

K.1a-4 Confirm that PHI will not be reused or disclosed to any other person or entity, except: as required by law, for authorized oversight of the research project, or for other

research for which the use of disclosure of PHI would be permitted by HIPAA.

✓ Yes

No

K.2 Will participant data and/or specimens be released outside of Lurie Children's?

Yes

✓ No

2 Study Setting(s)

A Study Setting(s)

A.1 Select all locations below where this study will be conducted.

✓ Ann & Robert H. Lurie Children's Hospital of Chicago and/or one of its outpatient centers.

Northwestern University or a Northwestern University Affiliate site (Prentice Women's Hospital, Northwestern Medicine, Shirley Ryan Ability Lab, Central DuPage Hospital, etc.)

School

Community Setting(s)

Pediatric Practice Research Group (PPRG); including those within/utilizing Community Connect.

Primary Care Physician Offices

Other

A.2 Is Lurie Children's serving as the IRB of record for any other study sites (e.g., Northwestern University, Prentice, Shirley Ryan, or other external sites)?

For more information on Lurie Children's serving as the IRB of record for Northwestern University, please visit the ORIC Website.

Yes, Lurie Children's will serve as the IRB of record for another site.

No, Lurie Children's is relying on an external IRB of record for this study.

No, each institution will conduct a separate IRB review.

✓ N/A, there are no other study sites

B Research Support Services

Select all support services that are being used for this study:

B.1 For more information regarding letters of support and other resources provided for Investigators and research personnel conducting clinical studies at Lurie Children's, please see the Clinical Studies Resources page.

Research Pharmacy -
The study involves preparation/storage or dispensing of medications or biologics.

Clinical Research Unit (CRU) -
The study requires CRU space and/or CRU personnel to support study visits.

Medical Imaging Research Committee -
The study requires deviation from the Medical Imaging standard of care imaging protocol and/or research imaging that is not being billed to patient insurance.

Research Laboratory (Department of Pathology and Laboratory Medicine) -
The study requires specimen, tissue, or slide processing, testing, storage, and/or shipping.

Cardiopulmonary Lab -
The study involves cardiopulmonary testing.

Infection Prevention and Control -
The study 1) utilizes equipment/instrumentation (provided or purchased) that will be used on more than one patient including items that requires sterilization/disinfection before first time use, and/or 2) involves the administration of a microorganism or a product that contains one or more microorganisms (i.e., bacteria, fungi, or virus; even if attenuated/non-replicable) to a participant.

Nursing Research Council -
The PI/Co-PI is a nurse or the subjects being studied are nurses.

Lurie Cancer Center Scientific Review Committee -
An oncology protocol that has not been reviewed by the NCI peer-review agency.

Emergency Medicine -
Studies that will directly recruit study participants from the Emergency Department (ED), or utilize ED services or staff for research purposes require written support from the Division.

Other internal or external divisions/departments/groups (Pediatric Intensive Care Unit, Ophthalmology, Anesthesia, etc).

✓ N/A; No support services used.

Safety Reviews

C

If any additional safety committee reviews are required for this protocol, the review and approval by these safety committees will be required before the IRB will issue a final approval. For more information, refer to IRB Policies and Procedures Manual Section 9.

Radiation Safety Review:

C.1 Does this study include ionizing radiation imaging exams (i.e., x-rays, DEXA, fluoroscopy, CT, nuclear medicine, and PET/CT scans)?

Questions regarding Radiation Safety and if review is required should be directed to the Radiation Safety Officer *(link to email)*.

Yes

✓ No

Institutional Biosafety Review:

Does this study involve the transfer into one or more human participants of recombinant or synthetic nucleic acid molecules; cells, organisms, and viruses

containing such molecules; (including Human gene transfer; Vaccine trials that involve the administration of recombinant or synthetic nucleic acid molecules)?

C.2

In the context of the *NIH Guidelines*, recombinant and synthetic nucleic acids are defined as:

I. molecules that a) are constructed by joining nucleic acid molecules and b) that can replicate in a living cell, i.e., recombinant nucleic acids;
II. nucleic acid molecules that are chemically or by other means synthesized or amplified, including those that are chemically or otherwise modified but can base pair with naturally occurring nucleic acid molecules, i.e., synthetic nucleic acids, or
III. *molecules that result from the replication of those described in (i) or (ii) above.*

Questions regarding Institutional Biosafety Committee and if review is required should be directed to IBC@luriechildrens.org.

Yes

✓ No

A Protocol and Consent Documents

Refer to the IRB Consent Forms & Resources Page for all informed consent templates and guidance for writing the documents.

A.1 Attach the main study protocol

IRBProtocol_v02_MatossianCommHealthGrant_20200910.docx

A.2 Attach the Lurie Children's parental permission forms to be used for this study

A.3 Attach the Lurie Children's adolescent assent forms to be used for this study

A.4 Attach the Lurie Children's adult consent forms to be used for this study

A.5 Attach the Study Information Sheets if a Waiver of the Requirement of Obtaining a Signed Consent Form was requested

2020-3640 CHNA_Information Sheet_English_CLEAN.docx

2020-3640 CHNA_Information Sheet_English_TC.docx

B Additional Supporting Documents

B.1 Attach Package Inserts or Investigator's Brochures for drugs/biologics and device label and instructions for use.

B.2 Study diaries

B.3 Miscellaneous Sponsor/FDA Documentation

B.4 Do any other documents require IRB review?

✓ Yes

B.4 Attach any other documents requiring IRB review.

BarriersSurveyEng.doc

No

Modification Submission

M1 Modification Information

A What type of submission is this?

✓ Modification

A Has the Lurie Children's IRB accepted the review of an external IRB for this study?

Yes

✓ No

Report of modification(s) implemented for immediate patient safety without prior IRB approval

Five day follow-up report after emergency use of a test article (drug, biologic or device)

Modification Description and Justification

Please refer to IRB Policies & Procedures Manual Section 9.2D.iv for details regarding Modification submissions.

A

Mark all items below being changed/updated/added/removed with this modification and thoroughly complete all follow-up questions.

Please remove tracked changes versions of documents that are not being revised with this modification from the application.

A.1 Study Title

A.2 Funding source(s)

A.3 Northwestern University or its affiliates involvement/engagement

A.4 Principal Investigator (PI)

✓ A.5 Study personnel

A.5a Provide the name(s) of the person/people being added/removed/changed in role.

adding Sara Malik to the study

Please ensure that this change is made in *Section 1.*

A.6 Study design and/or procedures and/or updated study protocol

A.7 Number of study participants

A.8 Study population or inclusion/exclusion criteria

A.9 Waivers of Consent and/or HIPAA Authorization

✓ A.10 Addition of foreign language translation of consent/assent or other documents

Please ensure that the approved consent forms, new translated versions, and corresponding Translation Certificate are uploaded in *Section 9.*

✓ A.11 Recruitment materials, verbal scripts, survey instruments, web-based instruments, questionnaires, etc.

A.11a Provide details of the change in recruitment materials and/or other study documents.

We have added "Patient Name" line to the upper righthand corner of the Barriers survey

A.11b Provide justification of the change in recruitment materials and/or other study documents.

Our study is hoping to correlate the survey responses to patient's clinical information. Originally we were going to identify the patients on the external surface of the envelopes which contained the Barriers survey. We decided we do not want PHI on the external surface of the envelope to prevent identifying the patient and to protect confidentiality, so we have added this to the survey itself.

Please ensure that these new documents are provided in *Sections 3, 4 and/or 9*.

A.12 New information about the investigational agent (i.e., updated Investigational Brochure)

A.13 Updated safety information or change in study status (i.e., DSMB/C or monitor report, or opening or closing of the study)

A.14 Planned protocol deviation

A.15 Expanded recruitment/enrollment to include persons from an European Economic Area (EEA) or sponsor notification of GDPR requirement

A.16 Other

B **Modifications Requiring Re-Consent of Active or Past Participants**
Please see IRB Policies & Procedures Manual Section 11.1K for guidance regarding the requirement for re-consent of previously enrolled subjects.

Have any participants been enrolled in this study at Lurie Children's?

Yes

✓ No

N/A - study does not enroll active participants.

1 General Information

Study Identification

Guidance for specific questions is included to the right of the question in the Helper Text (Question Mark Icon).

A

- Links to applicable sections of the IRB Policies & Procedures Manual are included throughout this application for reference.
- For more information about the IRB submission Process, IRB Tracking, and Cayuse IRB, please refer to the Cayuse IRB Submission Process guide.

What type of submission is this?

A.1

For details regarding the types of submission, please refer to IRB Policies & Procedures Manual Section 9.

Research Study Involving Human Subjects - **Expedited Review**
(Study involves procedures that are no more than minimal risk.)

Research Study Involving Human Subjects - **Full Board Review**
(Study involves procedure(s) greater than minimal risk or a minor increase over minimal risk; or the study includes an investigational device that is non-exempt and requires a Risk Determination.)

✓ Research Study Involving Human Subjects - **Exempt Determination Request**

Research Study Involving Human Subjects - **External IRB Review**
(i.e. request for Lurie Children's IRB to rely on an External IRB)

Treatment Use of Investigational Drug or Device
(i.e. Expanded Access, Humanitarian Use Device (HUD), Compassionate Use - Device)

Emergency Use of an Investigational Drug or Device

Use of Protected Health Information (PHI) Preparatory to Research

Case Report / Case Series

Quality Improvement / Quality Assurance Project

Use of Decedents' Protected Health Information (PHI)

Non-Human Subjects Research Determination

B

Study Personnel

For guidance regarding PI Responsibilities and who to list in Study Personnel, refer to IRB Policies & Procedures Section 5.

B.1

Select the Principal Investigator (PI).

Any study conducted by a PI who is not a Lurie Children's employee must have at least one Lurie Children's faculty member within the division/department where the research will be conducted serve as a Sub-Investigator.

Name: Debora Matossian
Organization: Kidney Diseases
Address: , Chicago, IL 60611-2605
Phone: 312-227-6165
Email: dmatossian@luriechildrens.org

B.2

Select all Primary Study Contact(s).

Name: Stephen Harris
Organization: Kidney Diseases
Address: , Chicago, IL 60611-2605
Phone:
Email: sharris@luriechildrens.org

Select all Sub-Investigators.

B.3 Name: Priya Verghese
Organization: Users loaded with unmatched Organization affiliation.
Address: , Minneapolis, MN 55455-2070
Phone:
Email: pverghese@luriechildrens.org

Select all other study staff.

B.4 If you are working with REDCap at Northwestern, please list "REDCap User" in this role so that REDCap staff may access to approval status and letters.
Name: Sara Malik
Organization: Un-Affiliated
Address: , Chicago, IL 60611-2605
Phone:
Email: saramalik@luriechildrens.org

B.5 Select non-Lurie Children's personnel engaged in the study conduct at Lurie Children's site(s).

B.6 To whom has the PI delegated responsibility to obtain informed consent?

All Primary Study Contact(s) listed in B.2

All Sub-Investigators listed in B.3

All other study staff listed in B.4

All non-Lurie Children's personnel listed in B.5

Limited to the following personnel:

✓ Not applicable (waiver of informed consent being requested or informed consent not required)

Exempt Determination Request

J

For guidance regarding studies exempt from IRB review, refer to [IRB Policies & Procedures Manual Section 9.2.b].

J.1 Select all categories into which this study most closely fits:

Category 1:
This research will be conducted in an established or commonly accepted educational setting.

It will involve normal educational practices that are not likely to adversely impact students' opportunity to learn required educational content or the assessment of educators who provide instruction.

The study procedures include:

- research on regular and special education instructional strategies; **or**
- research on the effectiveness of the comparison among instructional techniques, curricula, or classroom management methods.

✓ **Category 2:**
This research only includes interactions involving one or more of the following:

- educational tests (cognitive, diagnostic, aptitude, achievement)
- survey procedures
- interview procedures
- observation of public behavior (including visual or auditory recording)

J.1a Please select the interaction(s) to be included in this study.

Educational tests (cognitive, diagnostic, aptitude, achievement, etc).

✓ Surveys and/or interviews.

J.1a-2 Confirm that children will not be included as subjects in this study.

✓ Yes

No

Upload the surveys and/or interview scripts to be used for this study.

J.1a-3 CHNA_BarriersSurveyEng_11.06.20_CLEAN.doc

CHNA_BarriersSurveyEng_11.06.20_TC.doc

CHNA_BarriersSurveySpa_11.06_20.doc

Observation of public behavior (including visual or auditory recording).

J.1b Studies may be exempt under this category if at least one of the following criteria is met. Select all that apply:

The information will be recorded so that the identity of the participant **cannot** readily be ascertained (i.e. the study data will not contain identifiers and will not be linked to identifiers via a code).

The disclosure of the participants' responses outside the research would not reasonably place the participants at risk of criminal or civil liability or be damaging to the participants' financial standing, employability, educational advancement, or reputation.

✓ The information obtained is recorded so that the identity of participant **can** readily be ascertained (i.e. the study data is recorded with identifiers or identifiers are linked via a code).

Note: Studies involving Northwestern University Feinberg School of Medicine students or Northwestern/McGaw residents or fellows as study participants are to be reviewed and approved by Diane Wayne, MD, Vice-Dean, Education, Northwestern University Feinberg School of Medicine (dwayne@northwestern.edu).

Category 3:
This research involves benign behavioral interventions.

- The information will be collected from an **adult** participant through verbal or written responses (including data entry) or audiovisual recording; and
- The subject will be asked to prospectively agree to the intervention and information collection (i.e. written or verbal consent will be sought).

Benign behavioral interventions are brief in duration, harmless, painless, not physically invasive, not likely to have a significant adverse lasting impact on the participants, and the investigator has no reason to think the participants will find the interventions offensive or embarrassing.

Category 4:

✓ The secondary research use of identifiable private information or identifiable biospecimens.

J.1e Studies may be exempt under this category if at least one of the following criteria is met. Select all that apply:

The identifiable private information or identifiable biospecimens are **publicly available.**

The information will be recorded so that the identity of the participant **cannot** readily be ascertained (i.e. the study data will not contain identifiers and will not be linked to identifiers via a code).

In addition, the investigator will not contact the participants and the investigator will not re-identify participants.

✓ The information obtained is recorded so that the identity of participant **can** readily be ascertained (i.e. the study data is recorded with identifiers or identifiers are linked via a code). For example, collecting identifiable or coded data from the medical record.

This research is being conducted by, or on behalf of, a Federal department or agency using government-generated or government-collected information obtained for nonresearch activities.

Notes: Studies involving Northwestern University Feinberg School of Medicine students or Northwestern/McGaw residents or fellows as study participants are to be reviewed and approved by Diane Wayne, MD, Vice-Dean, Education, Northwestern University Feinberg School of Medicine (dwayne@northwestern.edu).

J.1h

Provide the source of the data and/or specimens:

- For example: Lurie Children's medical records, leftover clinical biospecimens, a research repository or database, Centers for Medicare & Medicaid Services database, etc.).

Lurie Children's medical records

Category 5:

This research /demonstration project is being conducted or supported by a Federal department or agency, or it is subject to the approval of a specific Federal department or agency/bureau or other subordinate agencies that have been delegated authority to conduct this project.

This research is designed to study, evaluate, improve, or otherwise examine public benefit or service programs, including:

- procedures for obtaining benefits or services under those programs;
- possible changes in or alternatives to those programs or procedures; or
- possible changes in methods or levels of payment for benefits or services under those programs.

(Projects applicable for this category include, but are not limited to, internal studies by Federal employees, and studies under contracts or consulting arrangements, cooperative agreements, or grants.)

Category 6:

Taste and food quality evaluation and consumer acceptance studies:

- if wholesome foods without additives are consumed; **or**
- if a food is consumed that contains a food ingredient at or below the level and of a use found to be safe, or agricultural chemical or environmental contaminant at or below the level found to be safe, by the Food and Drug Administration (FDA) or approved by the Environmental Protection Agency (EPA) or the food Safety and Inspection Services of the U.S. Department of Agriculture (USDA).

Briefly describe the study being proposed and provide the background and justification of the study including appropriate references if not included in a separate document/protocol in Section 9.

The intervention proposed is to evaluate the pediatric Hispanic population we serve in the Division of Kidney Diseases at Lurie Children’s Hospital.

The purpose of our study is to:
a)
Do a cross-sectional analysis of the pediatric Hispanic patient population of the Division of Nephrology at Ann & Robert H. Lurie Children’s Hospital. By means of a retrospective chart review of our pediatric Hispanic patients, we will analyze demographics (including zip code analysis and surrogates for socio-economic status); obesity/overweight rates, rates of adherence/follow up, presence of hypertension, distribution of kidney conditions they suffer from, glomerular filtration rate, proteinuria (the term Hispanic refers to all persons of Latin American origin living in the United States, unless indicated otherwise)
b)
Administer a simple questionnaire for patient families to evaluate the status of access to care for Hispanics; identification of unique issues of this population that precludes optimal follow up and care.

The primary study outcomes will be:
(1) To identify risk factors (disease specific and demographic) for progression of CKD in the Hispanic population served in the Kidney Diseases Division at LCH
(2) To perform a survey analysis of reported difficulties and limitations towards adequate access to care

Data to be collected:

• Zip Code
• Preferred language
• Age
• Gender
• Race
J.2 • Ethnicity
• Food insecurity
• Transportation needs
• Social documentation
• Financial resource strain
• Insurance
• Number of clinic visits within the study period
• Number of telemed / telephone / in person visits Following implementation of the telemed program at Lurie’s
• Number of hospitalizations within the study period
• No show rate
• Body mass index percentile (BMI = ht. in m2/wt. in kg.) for age and gender;
• Height and weight measurements
• Height and weight percentiles
• Most recent outpatient clinic BPs
• Clinical diagnosis to require clinical follow up at the Division of Kidney Diseases (primary + 4 additional from problem list)
• Renal replacement (Hemodialysis, Peritoneal dialysis, Transplantation, kidney transplant

waitlist)
• Concurrent serum cystatin C levels;
• Vitamin D levels;
• Serum glucose
• Total cholesterol, Triglycerides, HDL, LDL
• Hemoglobin A1C (if available)
• Urine protein/creatinine to calculate a ratio
• Serum creatinine
• Concurrent calculated eGFR

Survey:
-We will utilize a validated survey tool “Barriers to Care Questionnaire (BCQ)” in Spanish and English)\ to assess limitations to access to care.
-Survey will be completed by parents or patients older than 18 years old if they come by themselves.
-Paper survey tool will be handed to families at check-in.
- Survey will be voluntary.
-An information sheet will be attached to the survey.
- Survey will be placed in an envelope and will be collected by the research coordinator periodically.

List the inclusion and exclusion criteria for this study.

Inclusion criteria:
J.3 •Hispanic pediatric patients followed clinically in the division of kidney diseases at LCH
•Aged newborn to 21 years old

Exclusion criteria:
•Non-Hispanic patients

Provide the following:

- A plan to minimize any potential risks to participants,
- The process by which the privacy of participants and the confidentiality of the research data will be maintained; and
- A plan for monitoring the safety and welfare of enrolled participants.

J.4

All data will be stored in a secure password-protected database. All completed surveys will be stored in locked cabinets in locked offices.

Risk to subjects whose data will be collected is minimal. The primary risk is loss of confidentiality. As a retrospective chart review study, there is no plan for monitoring the safety and welfare of enrolled participants.

K Data Use/Disclosure

K.1 Will medical records or other sources of identifiable health information be accessed as a part of this proposal?

✓ Yes

K.1a Will the patient or patient's Legally Authorized Representative (LAR) be asked to sign a HIPAA Authorization Form for this proposed use of PHI?

Yes

✓ No - full waiver of HIPAA Authorization requested

K.1a-1 Confirm that the use or disclosure of PHI identifiers involves no more than minimal risk to the privacy of the participants.

✓ Yes

K.1a-2 Explain why this proposal cannot practicably be conducted without the Waiver of HIPAA Authorization to access and use the identifiers.

This is a retrospective review covering patients treated at Lurie Children's Hospital over a 3 year period.. If HIPAA authorization is required, it is likely that many patients would not be able to be contacted which would significantly negatively impact the quality of the data.

K.1a-3 Confirm that the PHI will be destroyed at the earliest opportunity.

✓ Yes

Confirm that PHI will not be reused or disclosed to any other person or entity, except: as required by law, for

K.1a-4 authorized oversight of the research project, or for other research for which the use of disclosure of PHI would be permitted by HIPAA.

✓ Yes

No

K.2 Will participant data and/or specimens be released outside of Lurie Children's?

Yes

✓ No

2 Study Setting(s)

A Study Setting(s)

A.1 Select all locations below where this study will be conducted.

✓ Ann & Robert H. Lurie Children's Hospital of Chicago and/or one of its outpatient centers.

Northwestern University or a Northwestern University Affiliate site (Prentice Women's Hospital, Northwestern Medicine, Shirley Ryan Ability Lab, Central DuPage Hospital, etc.)

School

Community Setting(s)

Pediatric Practice Research Group (PPRG); including those within/utilizing Community Connect.

Primary Care Physician Offices

Other

A.2 Is Lurie Children's serving as the IRB of record for any other study sites (e.g., Northwestern University, Prentice, Shirley Ryan, or other external sites)?

For more information on Lurie Children's serving as the IRB of record for Northwestern University, please visit the ORIC Website.

Yes, Lurie Children's will serve as the IRB of record for another site.

No, Lurie Children's is relying on an external IRB of record for this study.

No, each institution will conduct a separate IRB review.

✓ N/A, there are no other study sites

B Research Support Services

Select all support services that are being used for this study:

B.1 For more information regarding letters of support and other resources provided for Investigators and research personnel conducting clinical studies at Lurie Children's, please see the Clinical Studies Resources page.

Research Pharmacy -
The study involves preparation/storage or dispensing of medications or biologics.

Clinical Research Unit (CRU) -
The study requires CRU space and/or CRU personnel to support study visits.

Medical Imaging Research Committee -
The study requires deviation from the Medical Imaging standard of care imaging protocol and/or research imaging that is not being billed to patient insurance.

Research Laboratory (Department of Pathology and Laboratory Medicine) -
The study requires specimen, tissue, or slide processing, testing, storage, and/or shipping.

Cardiopulmonary Lab -
The study involves cardiopulmonary testing.

Infection Prevention and Control -
The study 1) utilizes equipment/instrumentation (provided or purchased) that will be used on more than one patient including items that requires sterilization/disinfection before first time use, and/or 2) involves the administration of a microorganism or a product that contains one or more microorganisms (i.e., bacteria, fungi, or virus; even if attenuated/non-replicable) to a participant.

Nursing Research Council -
The PI/Co-PI is a nurse or the subjects being studied are nurses.

Lurie Cancer Center Scientific Review Committee -
An oncology protocol that has not been reviewed by the NCI peer-review agency.

Emergency Medicine -
Studies that will directly recruit study participants from the Emergency Department (ED), or utilize ED services or staff for research purposes require written support from the Division.

Other internal or external divisions/departments/groups (Pediatric Intensive Care Unit, Ophthalmology, Anesthesia, etc).

✓ N/A; No support services used.

C Safety Reviews

If any additional safety committee reviews are required for this protocol, the review and approval by these safety committees will be required before the IRB will issue a final approval. For more information, refer to IRB Policies and Procedures Manual Section 9.

C.1 Radiation Safety Review:

Does this study include ionizing radiation imaging exams (i.e., x-rays, DEXA, fluoroscopy, CT, nuclear medicine, and PET/CT scans)?

Questions regarding Radiation Safety and if review is required should be directed to the Radiation Safety Officer *(link to email)*.

Yes

✓ No

Institutional Biosafety Review:

Does this study involve the transfer into one or more human participants of recombinant or synthetic nucleic acid molecules; cells, organisms, and viruses

containing such molecules; (including Human gene transfer; Vaccine trials that involve the administration of recombinant or synthetic nucleic acid molecules)?

C.2 In the context of the *NIH Guidelines*, recombinant and synthetic nucleic acids are defined as:

I. molecules that a) are constructed by joining nucleic acid molecules and b) that can replicate in a living cell, i.e., recombinant nucleic acids;
II. nucleic acid molecules that are chemically or by other means synthesized or amplified, including those that are chemically or otherwise modified but can base pair with naturally occurring nucleic acid molecules, i.e., synthetic nucleic acids, or
III. *molecules that result from the replication of those described in (i) or (ii) above.*

Questions regarding Institutional Biosafety Committee and if review is required should be directed to IBC@luriechildrens.org.

Yes

✓ No

A Protocol and Consent Documents

Refer to the IRB Consent Forms & Resources Page for all informed consent templates and guidance for writing the documents.

A.1 Attach the main study protocol

IRBProtocol_v02_MatossianCommHealthGrant_20200910.docx

A.2 Attach the Lurie Children's parental permission forms to be used for this study

A.3 Attach the Lurie Children's adolescent assent forms to be used for this study

A.4 Attach the Lurie Children's adult consent forms to be used for this study

A.5 Attach the Study Information Sheets if a Waiver of the Requirement of Obtaining a Signed Consent Form was requested

2020-3640 CHNA_InfoSheet_English_CLEAN.docx

2020-3640 CHNA_InfoSheet_English_TC.docx

B Additional Supporting Documents

B.1 Attach Package Inserts or Investigator's Brochures for drugs/biologics and device label and instructions for use.

B.2 Study diaries

B.3 Miscellaneous Sponsor/FDA Documentation

B.4 Do any other documents require IRB review?

Yes

✓ No
